# Optimisation of Response Surface Methodology Based on Finite Element Analysis for Laser Cladding of Highly Hardened WC(Co,Ni) Coatings

**DOI:** 10.3390/ma18153658

**Published:** 2025-08-04

**Authors:** Dezheng Wu, Canyu Ding, Mingder Jean

**Affiliations:** 1 College of Arts and Design, Jimei University, 185 Yinjiang Rd., Jimei District, Xiamen 361021, China; 200661000091@jmu.edu.cn; 2 School of Film and Animation, Xiamen City University, Qianpu South Road, Siming District, Xiamen 361000, China; dingcanyu@xmcu.edu.cn

**Keywords:** WC(Co,Ni), residual stress, laser cladding, temperature field, finite element analysis, response surface methodology

## Abstract

In the present work, the optimization of ceramic-based composite WC(Co,Ni) welds by laser cladding was carried out using response surface methodology based on finite element analysis. The heat distribution and temperature field of laser-melted WC(Co,Ni) ceramic coatings were simulated using ANSYS software, which allowed the computation of the distribution of residual stresses. The results show that the isotherms in the simulation of the temperature field are elliptical in shape, and that the isotherms in front of the moving heat source are dense with a larger temperature gradient, while the isotherms behind the heat source are sparse with a smaller temperature gradient. In addition, the observed microstructural evolution shows that the melting zone domains of WC(Co,Ni) are mainly composed of unmelted carbides. These carbides are dendritic, rod-like, leaf-like, or net-like, and are agglomerated into smaller groups. The W content of these unmelted carbides exceeds 80%, while the C content is around 1.5–3.0%. The grey areas are composed of WC, Co and Ni compounds. Based on the regression model, a quadratic model was successfully constructed. A three-dimensional profile model of the residual stress behaviour was further explored. The estimated values of the RSM-based FEA model for residual stress are very similar to the actual results, which shows that the model is effective in reducing residual stress by laser cladding.

## 1. Introduction

Laser cladding is an advanced surface modification technology. It involves adding specific cladding materials to the surface of a substrate. A high-energy-density laser beam is used to melt these materials. It also melts a thin layer of the substrate surface [1,2,3]. Compared to plating, spraying, electroplating, and vapour deposition, laser cladding has the advantages of low dilution, dense structure, good adhesion between the coating and the substrate, better selection of suitable cladding materials, as well as variations in particle size and composition, etc. [4,5,6,7,8]. There is, therefore, great potential for the application of laser cladding technology.

By laser tungsten carbide cladding of steel surfaces, the wear resistance of these components can be significantly improved, thereby extending their service life, reducing maintenance costs, and minimising downtime [9,10,11,12,13]. This process not only greatly improves the cutting efficiency, so that the cutting is smoother and more efficient, but also greatly improves the machining accuracy, so that the quality of the workpiece is reliably guaranteed. Also, the service life of the tool is also greatly extended, reducing the time and economic costs associated with frequent tool changes [14,15,16]. These tools are widely used in automotive, aerospace, mould, and other high-tech manufacturing industries [17,18]. As a result, cutting tasks of various metal materials can be excellently fulfilled.

Among them, cemented carbide WC is widely used in the manufacture of various cutting tools, such as turning tools, milling cutters, drills, boring tools, etc., which can efficiently machine a variety of metal materials [19,20]. Moreover, tungsten carbide is brittle compared to certain metallic materials. Its impact resistance and fracture resistance are relatively weak. When subjected to a large impact force or stress concentration, it can easily crack or even fracture, which affects its service life and safety, thereby limiting its application in some high-impact intensity environments. In addition, during the laser cladding process, a series of complex heat and mass transfer phenomena are generated, such as temperature-dependent phase transitions of the material, transient laser beam absorption, and reflection [21,22]. Hence, the large amount of residual stresses, deformations, and cracks generated in WC welds by laser cladding result in failure problems. However, residual stresses can adversely affect the dimensional stability, yield strength, fatigue strength, and corrosion resistance of clad specimens. Excessive stresses are likely to cause brittle damage, cracking, deformation, delamination, etc., during cladding or in service [23,24,25]. Therefore, the cracking of tungsten carbide on the surface of laser-melted steel is a tremendous challenge. However, the study of the mechanism of stress generation during laser cladding is particularly important for the improvement of laser cladding technology. It is required to propose a new method to reduce and eliminate residual stresses. Recently, a number of studies have been published by researchers that aim to overcome the above-mentioned drawbacks of the laser cladding process [26,27,28]. A way to solve the above problems is provided by the development of numerical simulation methods.

Finite element analysis (FEA) is a powerful numerical analysis method, which has many advantages in the field of laser cladding, such as simulating the temperature and stress fields, optimising the process parameters, characterising the structure and properties of the cladding layer, analysing the effects of multi-physical coupling, reducing the cost and cycle time, as well as guiding the design of the cladding layer [29,30,31]. Researchers have increasingly emphasised the application of numerical simulation in the study of the temperature field in laser cladding. These studies have laid groundwork for the further development of numerical simulation of coating by laser cladding. They also show that the influence of the heat source model on the calculation results requires further in-depth study. In view of the above, the temperature field of the laser-melting process under the heat source model is calculated using the ANSYS APDL software with WC-Co-Ni welds. The calculated results are then compared with experimental results to improve simulation reliability [32,33,34,35]. However, the action of a laser on a metal surface is usually characterised by a numerical simulation of the temperature field, which is modelled as a heat source. The control equation for heat transfer is based on the principle of energy conservation. This equation is solved by the finite element methods using finite element software (ANSYS R15) [34,36,37]. By accurately simulating the physical field coupling and material behaviour during the cladding process, it enables process optimisation, defect control, and performance improvement.

As mentioned above, there are already several reports on finite element studies of the mechanical properties of WC(Co,Ni), but the results of residual stress obtained by numerical simulation can only be used to explain some complex physical phenomena of the coating layer in laser cladding. Accordingly, it is incapable of solving the problem of optimising the process parameters in the experimental domain, thereby failing to address the relationship between each parameter and the residual stresses, as well as their significance [27,28,29,30,31,32,33,34,35,36,37]. However, the residual stresses in the cladding layer are mainly due to the thermal stresses during the cladding process. It is necessary to have a better understanding of the mechanism of finite element analysis on the mechanical properties of laser-clad WC(Co,Ni) coatings. Therefore, it is particularly important to determine how to select the parameters of the optimal process in the model simulation of finite elements so that favourable residual stress distributions in the cladding deposit can be obtained. Therefore, it is necessary to combine finite element simulation with the response surface methodology, which is an alternative solution.

Response surface methodology (RSM), which can be used to optimise extraction, is a group of mathematical and statistical techniques that are widely used in the manufacturing industry to evaluate the relationship between predicted values of independent and dependent variables [38,39]. The primary advantage of RSM is that it reduces the number of experiments required. This is to evaluate multiple variables and their interactions. Consequently, RSM is a more efficient use of time and effort than other methods. Several studies have demonstrated the effectiveness of RSM in investigating optimal extraction conditions for the mechanical properties of welding [40,41,42,43,44]. However, integrating FEA and RSM is an alternative, and there is no information available on the optimization of RSM based on finite elements for WC(Co,Ni) welds.

In this study, a blended WC(Ni,Co) powder was deposited by laser cladding. A 3D finite element simulation of the temperature and stress fields during the laser cladding process was carried out using the ANSYS software package. A model of the metal–ceramic composite weld was constructed using RSM combined with finite element analysis. The laser parameters were then optimised to obtain reduced residual stress characteristics of the highly hardened WC. In addition, the residual stress behaviour, microstructural evolution, and fracture crack formation of WC deposits with different Co/Ni ratios in composite welds by laser cladding were investigated. The results not only provide optimised process parameters for laser welding of WC(Co,Ni) welds, but also clarify the correlation between process parameters and residual stresses in the experimental domain. This will help to make more reasonable and appropriate adjustments in the selection of process conditions and optimisation of quality control, thereby significantly improving production efficiency.

## 2. Experiments and Methods

### 2.1. Materials and Preparations

**Figure 1:**
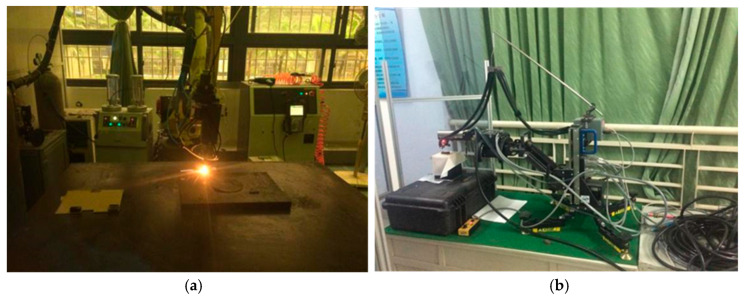
(**a**) A fibre laser cladding system. (**b**) The tester for residual stresses of Proto i-XRD devices.

**Figure 2:**
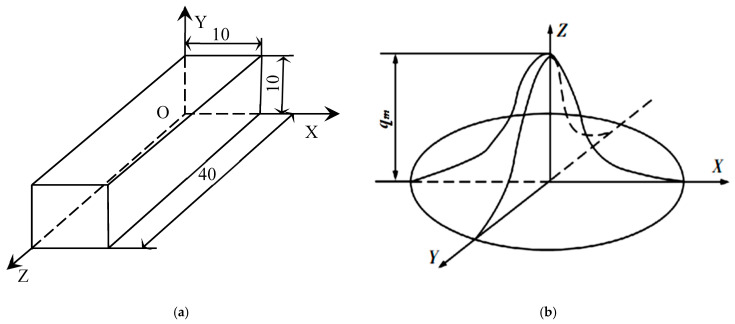
(**a**) Dimension of the model construction. (**b**) Schematic of the heat source model for the Gaussian surface.

The fibre laser cladding system represents an advanced surface treatment technique. The laser cladding system consists of a fibre laser, a six-axis robot, a powder feeder, an induction power supply (HUST III, Mitsubishi, Tokyo, Japan), a computerised digital control system, and a cooling system. The device was manufactured by IPG YLS-3000 fiber optic laser in Burbach, Germany. By utilising a fibre laser as a heat source, surface modification is achieved by melting materials with specific functions on the surface of the substrate that enhance the surface properties of the substrate. Figure 1a shows the schematic diagram of coxial powder delivery by laser cladding. The alloy powder is driven by the nozzle and shielding argon gas to flow through the powder feeder, which is heated by a laser. It enters the liquid melt pool on the surface, thereby solidifying and forming the cladding layer. An IPG YLS-3000 fiber optic laser wass used. It has a maximum power of 3000 W and a wavelength of 1.07 μm. The powder feeder of the HUST III is used to feed metal powder to the cladding area. The powder is usually blown by an air stream to the position where the laser beam is to be irradiated. WC(Co,Ni) blends consist of 125 lm to 300 lm of WC, Co, and Ni alloy powders. In addition, the analysis of residual stresses is carried out on the centerline of the weld channel using the sin2ψ method. By tilting the sample to change (ψ), it is equivalent to measuring the strain in different directions in which the variation in the grain spacing is measured to calculate the stresses. For this experiment, the Proto iXRD diffractometer was used with fixed diffraction at 0° and 90°. The Proto iXRD diffractometers were obtained from Proto Manufacturing Inc., Taylor, MI, USA. These data were obtained by tilting the sample stage with the sample at tilt angles of 0°, 15°, 30°, 45°, and 60°, where the diffraction peaks were collected at each angle. As shown in Figure 1b, an X-ray diffraction (XRD) is a portable or benchtop XRD instrument based on Bragg’s law that calculates stresses by measuring lattice strains at different diffraction angles using the Proto iXRD device. Also, the microstructure of the WC(Co,Ni) welds was analysed using a scanning electron microscope (SEM) with a Hitachi S-2600H, Tokyo, Japan. As shown in Figure 2a, the work metals are #45 steel and #40Cr steel with dimensions of 40 mm × 20 mm × 10 mm. The Taguchi method uses orthogonal arrays to systematically vary control factors and quantify their impact on response variables (e.g., residual stress). The factors and levels that were controlled in this study are shown on the left-hand side of Table 1, which is part of the orthogonal table. The values on the right side of the table are the residual stresses on the surface of the coating. As shown in Table 1, a total of 18 experiments were conducted using the orthogonal table of the Taguchi method. These experiments included one parameter with two levels and seven parameters with three levels. Each experiment was repeated three times to check the reproducibility of the results. By experimenting design with a large number of variables in the system, process variations can be significantly reduced. Due to the residual stress characteristics, and the smaller and better the type, the better the quality of the product. The present study employs ANSYS software to create a model of laser cladding. There are modules that cover material properties, mesh partitioning, Gaussian heat sources, boundary conditions, stress cloud maps, and temperature distribution maps. As illustrated in Figure 2b, a model of a Gaussian surface is used as the heat source to perform a numerical simulation of the temperature field. On the other hand, an RSM was constructed using the Taguchi method based on the finite element method. The residual stress of the laser cladding layer was simulated using a model, whose predictor was validated through experiments.

### 2.2. Finite Element Models

Laser cladding is a metallurgical process with rapid melting and solidification. The process offers great advantages in terms of characterising materials. However, laser cladding is a complex thermodynamic process. It is difficult to determine the changes in various parameters. This makes numerical simulation methods a good fit for this field. In recent years, many models have been developed to explain the heat transfer and flow processes in laser cladding. The accuracy of the transient welding temperature field is strongly influenced by the choice of the heat source model. This is especially true in the vicinity of the heat source. A Gaussian distribution of the heat source is often used for laser cladding, where the heat distribution is

(1) $$q_{r}=q_{m}\exp(-3\frac{r^{2}}{R^{2}})$$

where *q_r_* denotes the density of the heat source, r is the distance from the centre point of the spot, R is the radius of the spot, and *q_m_* is the maximum value in the heat flow density. The parameters of the material, both thermal and mechanical, are given for numerical finite element simulation using ANSYS [28]. A Gaussian heat source is a commonly used model. It is used for heat source distribution. The heat flow density of a Gaussian heat source is Gaussian distributed in space. The heat flow density of a Gaussian heat source model commonly takes the form of the following formulas:

1. The heat flow density q(x,y,t) of a Gaussian heat source in a two-dimensional plane, assuming that the heat source is moving along the z-axis with velocity v, is given by the following equation:

(2) $$q(x,z,t)=\frac{2Q}{\pi R_{0}^{2}}exp\left[-(\frac{2{\left[(x-x_{0}\right)}^{2}+{\left(z-vt\right)}^{2}]}{R_{0}^{2}\;})\right]$$

2. The equation for the heat flow density q(x,y,z,t) for a moving Gaussian heat source in three dimensions, assuming that the heat source is moving along the z-axis with velocity v, is:
  (3) $$q\left(x,y,z,t\right)=\frac{3Q}{\pi R_{0}^{2}h}exp\left[-(\frac{3{\left[(x-x_{0}\right)}^{2}+{\left(y-y_{0}-h\right)}^{2}+{\left(z-z_{0}-vt\right)}^{2}]}{R_{0}^{2}\;})\right]$$
  where q(x,y,z,t) is the density of heat flow, Q is the total laser power, R_0_ is the radius of the laser spot, v is the moving speed of the heat source, h is the depth of the heat source, t is the time, and x, y, and z are the coordinates in space. In this study, WC(Co,Ni) composite coatings were used in simulations and experiments.

**Figure 3:**
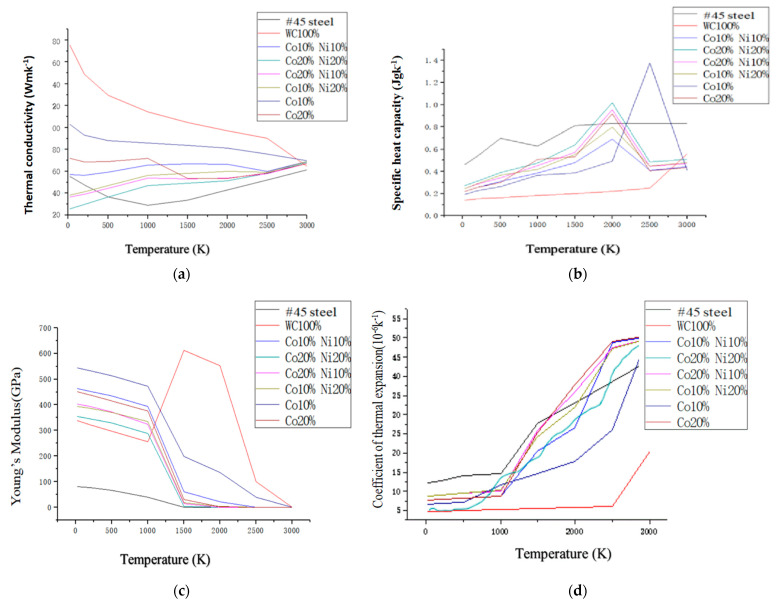
Thermophysical properties of various powders and #45 steel as a function of temperature (**a**) Coefficient of thermal conductivity. (**b**) Specific heat capacity. (**c**) Young’s modulus. (**d**) Coefficient of thermal expansion.

**Figure 4:**
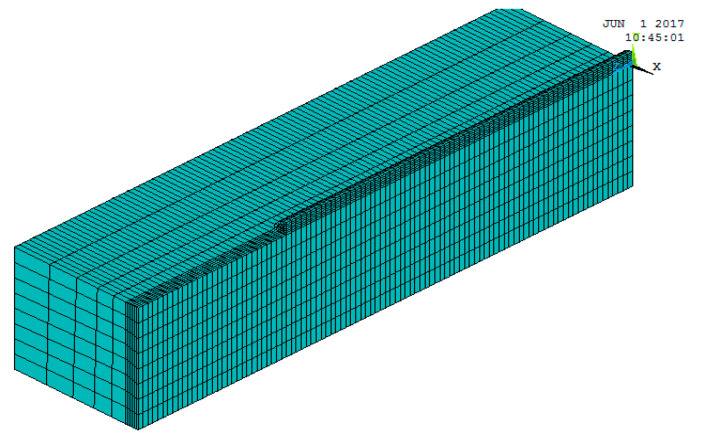
The cells activated at t_1_ sec to be loaded with the laser heat source.

All of the thermal and mechanical properties are considered as a function of temperature. This is performed in the model. This allows for more accurate results. The calculation of the temperature field during laser cladding involves problems of transient heat transfer that are non-linear. This is because the thermal conductivity and specific heat capacity of the material change with temperature. Therefore, the eigenvalues of material properties at several points of special temperature are determined in the calculation. Then the eigenvalues at other points of temperature are calculated according to the rule of change in the eigenvalues of the material by using the difference method. The variations in thermal conductivity, coefficient of linear expansion, specific heat capacity, and modulus of elasticity with temperature for various materials in the coating are shown in Figure 3. These materials include #45 steel and powder ratios. The model is presented with symmetry considered. The choice of a half section along the cladding direction is determined. This is to reduce the amount of computation. The dimensions of the model in this study are 40 mm × 10 mm × 10 mm as in shown Figure 4. The cross-section of the coaxial coating is a quarter-circle with a radius of 1 mm. The material unit type is defined as solid70 3D thermal solid unit. The number of model cells for the synchronous cladding layer is 2700 (27 × 100), and the number of matrix cells is 8800 (11 × 100 × 8). The ANSYS model is realised through the operation of the GUI interface. This generates a command flow. This facilitates the subsequent modification of the material parameters and the heat source model settings, etc. This operation generally appears at the beginning of the programme, thereby effectively improving work efficiency. ANSYS uses APDL. This is to program the movement process of the heat source. It uses the Cartesian coordinate system. In this system, the load is applied in the local coordinate system. It achieves the transformation from time to space. The transformation is performed by the coordinate transformation. This is in the direction of cladding, that is, in the direction of the O-Z axis. Meanwhile, the growth process of the cladding unit is simulated by using the technique of “live and dead” in ANSYS [2]. The relevant programme for the APDL is as follows:
ESEL, S,MAT,, 2
EPLOTKilled all the unitsEKILL, ALL
EPLOT


EPLOT
EALIVE, ALL
CM,E_1, ELEMActivation of units at the scan of the laserCM,N_1,NODE


### 2.3. Response Surface Methodology

In this study, many controlling factors are involved in the residual stresses in WC/Co/Ni welds during laser cladding as shown in Table 1. Therefore, RSM was used as an alternative method to analyse the properties of residual stresses, which is performed based on the RSM developed by Taguchi [39]. An ANOVA table containing quadratic terms was created based on regression function. The detailed statistical analyses were carried out using the SPSS 22 software package. A quadratic polynomial model was defined to fit the response to the following questions. The quadratic model of the stress response, Y, with the independent variable X is shown in Equation (4).

(4) $${Y=a}_{0}+a_{1\;}X_{1}+a_{2}X_{2}+a_{3}X_{3}+a_{4}X_{4}+a_{12}X_{1}X_{2}+a_{13}X_{1}X_{3}+a_{14}X_{1}X_{4}\;+a_{23}X_{2}X_{3}+a_{24}X_{2}X_{4}+a_{34}X_{3}X_{4}\;+a_{11}X_{1}^{2}+a_{22}X_{2}^{2}+a_{33}X_{3}^{2}+a_{44}X_{4}^{2}$$

where Y is the predicted response: residual stress. a_0_ is the is constant coefficient, a_1_, a_2_, a_3_ and a_4_ are linear effects, a_11_, a_22_, a_33_, and a_44_ are quadratic effects, and a_12_, a_13_, a_14_, a_23_, a_24_, and a_34_ are interaction effects. X_1_, X_2_, X_3_, and X_4_ are study factors.

## 3. Experimental Results and Discussion

### 3.1. Validation of Models in Finite Element Analysis

**Figure 5:**
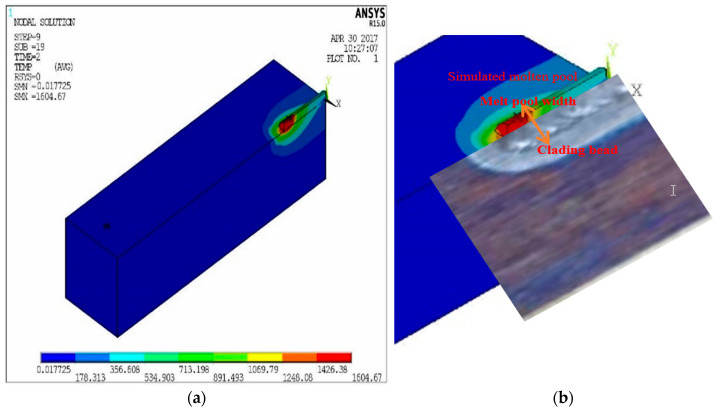
(**a**) Melt pool dimensions obtained from simulations at trial 9. (**b**) Local enlargement of the molten pool, where the width of the simulated molten pool is compared with the actual weld bead of the laser welding.

To establish that a simulation model was available, a series of simulations were carried out using various physical parameters detailed in Figure 3, in which the simulated stress results were compared with the experimental data. Due to the accurate modelling of the heat of the coatings during the laser process, the model is able to calculate reliable predictions of the thermal stresses by forming a temperature gradient in the resulting thermal cycle according to the above Equations (2) and (3). Figure 5a shows a detailed schematic of the profiles of the molten pool that were measured by the simulation model. Trial 9 of the group of 18 shown in Table 1 is an example chosen for comparison purposes, illustrating the behaviour of a typical molten pool under the conditions. The results show that the melt pool is in the shape of a comet. It has an elliptical front and a stretched tail. This indicates a wide front and narrow front of the melt pool width. The figure shows one of the 18 sets of simulation results in this study. It also shows a typical example of temperature change. This temperature change occurred during the movement of the heat source in the experiment. This can be used as a case study. It can be used for the experimental confirmation of the model. A localised enlargement of the molten pool is shown in Figure 5b, where the width of the simulated molten pool is compared with the actual weld of the laser weld. Three-dimensional modelling of the laser cladding process is performed. A Gaussian-distributed heat source is used. This yields cladding shapes. These shapes are in good agreement with experimental results for weld beads.

### 3.2. Validation of Temperature Field in Finite Element Analysis

**Figure 6:**
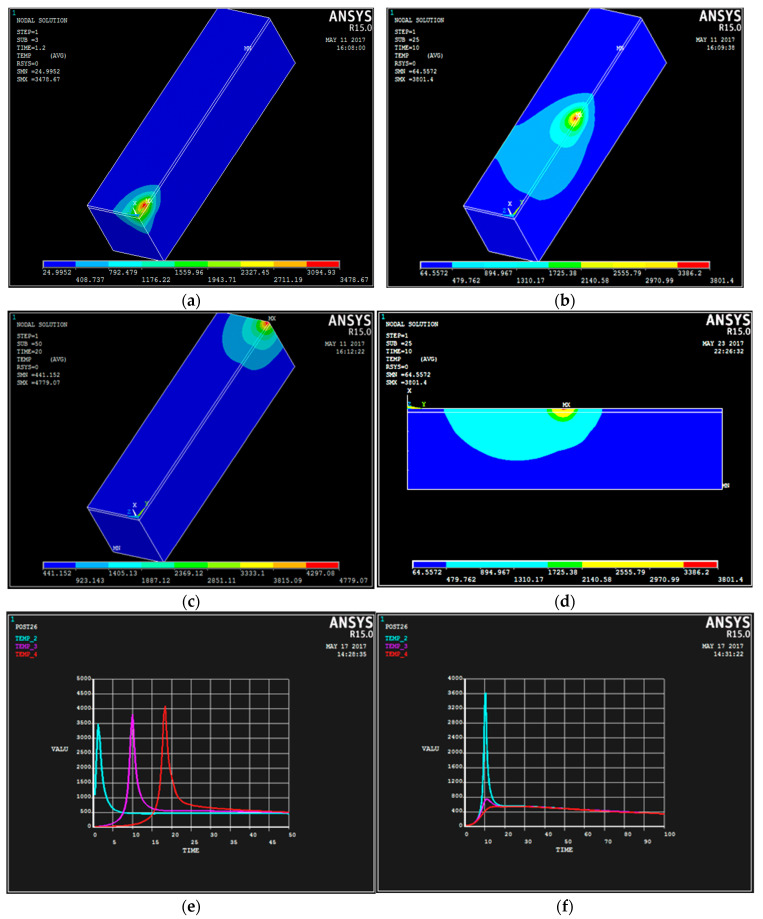
A view of the temperature field for the 5th trial at 1 s, 10 s, and 20 s. (**a**) The cloud of temperature field at time 1 s. (**b**) The cloud of temperature field at time 10 s. (**c**) The distribution of temperature field at 20 s. (**d**) The cloud of temperature field above the symmetry plane at 10 s. (**e**) Temperature–time profile along the direction of the welding track. (**f**) Temperature–time profile along the direction of the perpendicular melting track. The maximum temperature of the molten pool of the group is 4779 K and the minimum temperature is 3478.78 K.

The simulation was carried out using the process parameters of trial 5 in Table 1, similar to the study of Song et al. [29]. This is shown in Figure 6 for 1 s, 10 s, and 20 s along the direction of the laser beam. As can be seen, the temperature rises extremely rapidly, reaching maximum temperatures of 3478 K, 3801 K, and 4779 K, respectively, which is above the melting point at which WC(Co,Ni) and #45 steel melt together due to photoimpact. This leads to the formation of a molten pool. The molten pool is ellipsoidal, in which the highest temperature occurs behind the centre of the molten pool. In the early stage of cladding, the flow rate of the melt pool is low, and the energy transfer in the melt pool is by heat conduction, while with the progress of cladding, the molten metal in the molten pool is mainly convected by heat. Figure 6a,c show the temperature field distribution at different times, and the order of melting is from top to bottom. Figure 6a shows the temperature field distribution at the beginning of the melting for 1 s, in which the temperature field is in the front half elliptic shape like a comet; The distribution of the temperature field at 10 s after the laser travelled forward is shown in Figure 6b. The temperature field is a large ellipsoid, similar to a zucchini bag that is dragged backward. The maximum temperature of the dark green area inside the bag (3801 K) is compared with the minimum temperature of the light green area (894 K). The WC(Co,Ni) in the light source area has been melted, and the temperature difference between the melting area in the gourd bag is very large and decreases rapidly. Figure 6c shows the distribution of the temperature field. This is at 20 s after the coating is finished. The temperature field is in the shape of a back-dragging partial ellipse. The temperature field of the ellipse is symmetrical to the centre of the spot. In addition, Figure 6d shows the distribution of 2D temperature field at 10 s. As can be seen, the temperature in the middle of the workpiece is higher than in the surrounding area, forming a closed ring. The red zone is larger than 4439 K, the average temperature in the light-green zone is 1788 K, and the average temperature in the dark-green zone is 897 K. This shows that the temperature field is in an unstable state, i.e., the cladding layer is subjected to rapid cooling. As shown in Figure 6e, the temperature change process along the direction of the weld bead changes drastically with the time, at 1 s, 10 s, and 20 s, while the temperature of the three changes exceeds the melting point of the substrate at 750 K. Based on the welding order, the maximum temperature of the curve increases gradually from the green line to the pink line and then to the red line. When the laser spot enters the green, pink and red lines, respectively, the temperature of each increases rapidly. When the laser spot moves out, the temperature decreases rapidly. The pattern is apparently consistent. It can be seen that laser cladding process shows a rapidly heating and cooling behaviour. This is mainly due to the heat build-up in the material as it continues to absorb the laser input. In addition, the node temperature decreases steadily. It approaches 1000 K. This is mainly due to the phase transformation of the liquid metal. The liquid metal solidifies slowly. This causes the overall temperature to change relatively slowly. Whilst heating is forced during the warming process, the trend is less pronounced. Figure 6f shows the temperature–time curves perpendicular to the direction of the melting track. The temperature profiles at 1 s, 10 s, and 20 s, respectively, are consistent. The maximum temperature at 1 s (green) is much larger than those at 10 s (pink) and 20 s (red). The laser heat source passes through the surface. It also passes through the inner (vertical) layer at the location of the node. The temperature reaches a maximum value. These are 3800 K, 800 K, and 400 K, respectively. As the heat source approaches the surface of the coating, the temperature rises. This is mainly due to the fact that the energy of the heat source on the upper surface is more concentrated, and relatively more energy is absorbed. The temperature at the surface is higher than the melting point, while the temperature underneath is lower than the melting point, indicating partial melting of the powder along the thickness. These may give an insight into the properties of the melt pool, the heat-affected zone, and the substrate in the cladding. However, the temperature distribution at the three selected points (A, B, and C) is similar, while the temperature increase rate is slightly higher than the temperature decrease rate.

### 3.3. Evaluation of Residual Stress

**Figure 7:**
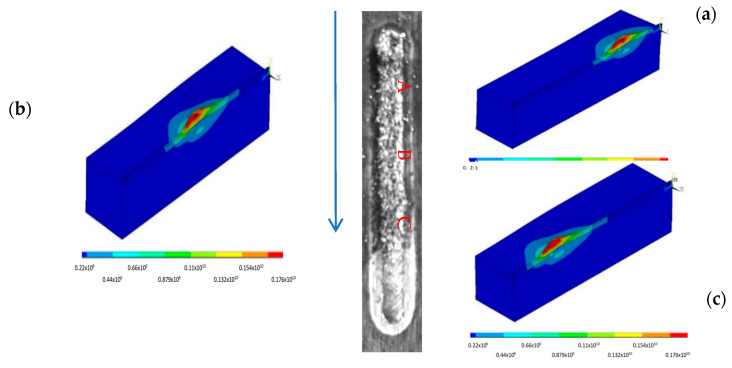
Distribution of stress at the Z-axis weld when the moving heat source (**a**) is at point A, (**b**) is at point B, and (**c**) is at the position indicated by C.

**Figure 8:**
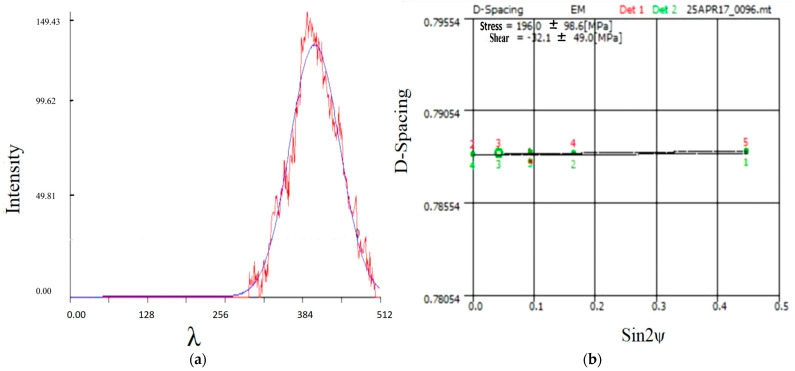
(**a**) The measured residual stresses of the pattern of fitting a Gaussian function to a peak measured by X-ray diffraction. (**b**) Value derived from the slope of the calculation of the lattice spacing versus sin 2ψ.

The nature of the coatings produced by the laser process makes the generation of residual stresses invetiable. This is due to high thermal gradients. These are coupled with high cycles of melting and solidification. To analyse the formation of residual stresses, we have carried out single-track simulations using the conditions of trial 1, based on model validation of the maximum temperature and melt pool size. Figure 7 shows the stress distribution of the model at different positions (A, B, and C) along the z-axis welding bead. The stresses at position A of the welding zone are shown in the upper right corner of the model, and the stresses at positions B and C are shown in the following order. It can be seen that the red area of the welding bead along the z-axis is more stressful. Various positions of the z-axis are modelled to show the stress distribution when the melt is cooled down to room temperature. It can be seen that the red area of the welding bead along the z-axis is more stressful. This is because the material is heated by the laser along its path (z-axis), which results in the expansion of the material. The values of their stresses range from 162 to 183 MPa (Figure 7a), 176 to 198 MPa (Figure 7b), and 168 to 189 MPa (Figure 7c), respectively. In addition, the areas on both sides of the z axis show rings of green and dark green, which have the values of 20.3–60.9 MPa, 22–66 MPa, and 21–63 MPa, respectively. A comparison of the above results shows that the residual stresses along the z-axis (laser scanning direction) are more noticeable than in the other two directions. However, the red area that was heated by the laser is rapidly cooled and contracted when the laser is moved forward, which generates high stress. This is mainly due to the fact that the xy-direction of the material is limited by the surrounding cooler material, so it is not free to expand or contract. Due to the influence of the laser movement, it leads to higher stresses in the z-direction. The x and y directions, on the other hand, are not directly affected by the rapid heating and cooling of the laser beam. As a result, they exhibit less thermal expansion and contraction compared to the z-axis. Accordingly, the residual stresses along the xy-axis are lower. To further validate the model of residual stresses, the surface of the cladding was examined for residual stresses using an IXRD type apparatus for residual stress measurement. The surface residual stresses of trial 1, determined by X-ray diffraction, were measured for three different combinations of WC(Co,Ni) components, as shown in Figure 8a,b. Figure 8a shows the X-ray diffraction results of one of the measured peaks fitted to a Gaussian function. Through the fitting operation of the measured peak, it could accurately extract the information of various parameters related to this peak. Figure 8b shows the residual stresses derived from the slope of the plot of lattice distance spacing (d-spacing) versus sin2ψ for five diffraction anglesψ. Further calculations and analyses of these parameters shown in Figure 8b make it possible to derive a numerical value of the residual stress in the material, thereby enabling an accurate calculation of the residual stress on the surface of WC(Co,Ni). The values of 196 MPa were measured as mentioned above. The three predicted values of A, B, and C for WC(Co,Ni) after modelling in this study are 189 MPa, 183 MPa, and 198 MPa, respectively. The mean value is 190 MPa. The results above show that the simulated values of the model are very similar to the experimental values. Therefore, the proposed model is reliable.

### 3.4. Evolution of Microstructure in the WC(Co,Ni) Welds

**Figure 9:**
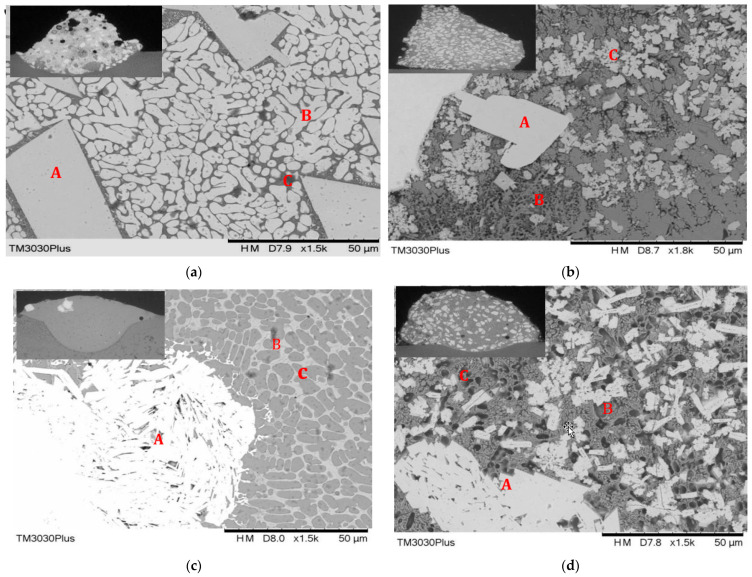
SEM microstructures of various tests with WC/Co/Ni deposits by laser cladding including (**a**) trial 7, (**b**) trial 9, (**c**) trial 14, (**d**) trial 17.

As shown in Figure 9, the structural features of the Co/Ni added WC weld are shown in SEM, which shows the etched structure of the weld as well as the microstructural evolution of the WC composite material. Also, a cross-section of the weld bead is shown in the upper left corner of Figure 9. The resulting cross-section shows a highlighted WC/Ni/Co deposit, which shows the molten zone, the heat-affected zone, and the zone of the substrate. Figure 9a shows trial 7 of Table 1, which is the morphology of the 20% Co + 80% WC melt pool. It exhibits a large mass of dissolved white carbides surrounded by a large portion of fine, elongated carbide structures. The microstructure consists mainly of tungsten carbide-rich blocky and dendritic solid solutions, with metallurgical defects and some unmelted tungsten carbide particles. In addition, Table 2 shows the EDS of three different locations (A, B and C) of the WC mixture in Figure 9 which is labelled as WC, Co and Ni atoms. There are highly unmelted tungsten carbide particles in the melt pool region, which has 94.43% W in region A. The surrounding area of very hard tungsten carbide particles that have high stress concentrations is likely to lead to the growth of cracks during the rapid solidification process. Some pores can be seen in the upper left section of the melt pool in trial 7. This is related to the fact that these pores contribute to stress release; in addition, the melt in the B and C regions contains close to 0.25% Co particles in the melt pool region. This is due to the good wettability of the molten Co-based alloy which appears to wet the carbide particles in this region. As mentioned above, it is known that the porosity and wettability of carbide grains can inhibit cracking [22], thus reducing the possibility of it happening. In trial 9, white carbides with some large particles, eutectic small massive carbides, and grey oxides were detected. As shown in the WC/Co/Ni weld in the upper left corner of Figure 9b, the white WC particles in the melting zone of trial 9 were uniformly distributed. No pores or cracks were found in the cross-sectional image of the coatings. There is a low degree of dilution, good metallurgical bonding, and few metallurgical defects. The microstructure of the weld by laser cladding is shown in Figure 9b. A large number of white carbides can be seen. These are found in region A. There are also leaf-like carbides in region B. Grey areas are found in region C. As shown in Table 2, in area A, the massive carbide is rich in C, Co, and Ni. The contents of C, Co, and Ni are much lower. The content of W is much higher than the others, at 88.85%. Most of the grey areas in zone B contain turbid grey oxides and coarse particles of molten tungsten carbide, which have lower W content but higher O and Fe levels. These areas contain much less than 25% W, 32.08% Co, and 27.92% Ni, which are clearly visible. The effect of cobalt-nickel/base alloys on stress is greater than that of WC. The C region precipitates leaf-shaped carbides containing more Co and Ni elements that solidify to form M(Co,Ni)_6_C, M(Co,Ni)_12_C, and M(Co,Ni)_7_C_3_ compounds. These compounds inhibit crack initiation and reduce susceptibility to cracking. Overall, there is a high stress generation in this region with a value of 265.21 MPa, but no cracks detected. This is mainly due to the fact that the compounds in this region are resistant to crack initiation, and the susceptibility to cracking is reduced. As shown in Figure 9c, the observation of the cross-section at the upper left corner in trial 14 shows that the tungsten carbide particles were almost completely melted. The melted tungsten carbide was found to be loose, with a small amount of dendritic carbide in area A. The EDS corresponding to Table 2 shows 89.98% W, 11.11% Co, and 11.95% Ni, with small amounts of C, O, and Fe elements. These structures may be due to the melting of cobalt/nickel in tungsten carbide, since they are the binding phase of tungsten carbide-based cemented carbides. The networked white carbides of the B-area are rich in 4.09% C, 33.36% Fe, 5.98% O, 11.49% Co, and 6.49% Ni content. They also contain a significant amount of 38.02%W. Similarly, the carbide content in zone C is similar to that in zone B. Only W decreases to 30.01%, while Ni increases to 9.6%. It exhibits a lower stress level of 96.32 MPa. This is mainly due to the active diffusion of Co and Ni atoms in the molten state. There is a significant effect on the crystallisation process of WC. This results in the formation of a network-like structure of WC. As shown in the upper left weld in trial17 of Figure 9d, the structure of the welded bead is uniformly distributed with white particles in the melting zone. Zone A contains coarse particles of finely melted WC, which appear loosely structured, and are clearly visible at a W value of 91.86%, but there is a small number of Co and Ni particles, approximately 2.5%. The distribution of W is 84% in region B, where the smaller clustered carbides approximate the structure of region A. The W content is reduced by only 3.46% Ni and 9.02% Co. The melting zone shows a decrease in W content, while the 9.02% Co element increases considerably. Based on the results of the EDS in area C, the W content in the weld bead is minimal among the three areas, which is about 46.27%. Meanwhile, the content of O (oxygen) in the weld reaches 12.66% and Co (Cobalt) is 28.9%, which shows a sharp increase in the content of these two elements. In contrast, the increase in Ni (Nickel) and C (Carbon) in the weld beads is relatively small. The effect of Ni-based alloys on WC is likely to be smaller than that of Co-based alloys. It exhibits higher stress, at 261.36 MPa, but no cracks were generated. This may be due to the favourable Co and Ni ratios. These form new carbide phases. These phases can affect the interfacial properties between WC and the binder phase. The binder phase enhances the binding force between them. It also inhibits the growth of cracks.

**Table 2:** Chemical composition of the WC(Co,Ni) particles, interface, eutectics, and dendrites in the cladding beads marked location in Figure 10 by EDX analysis.

No. of Trials		W	C	O	Fe	Ni	Co
Trial7	A	94.43	2.08	1.87	1.17	0.00	0.068
	B	92.08	1.53	3.12	2.81	0.00	0.28
	C	90.74	1.32	4.37	2.93	0.00	0.25
Trial9	A	88.85	1.61	1.08	3.12	2.89	2.15
	B	25.00	2.34	6.49	5.84	27.92	32.08
	C	66.22	1.93	4.48	3.76	11.95	11.11
Trial 14	A	89.98	2.97	1.54	2.42	1.479	0.99
	B	38.02	4.09	5.98	33.36	6.49	11.49
	C	30.01	4.99	7.14	36.07	9.60	11.30
Trial17	A	91.86	1.55	1.03	0.53	2.32	2.49
	B	84.00	0.99	1.74	0.71	3.46	9.02
	C	46.27	1.74	12.66	3.37	5.81	28.91

**Figure 10:**
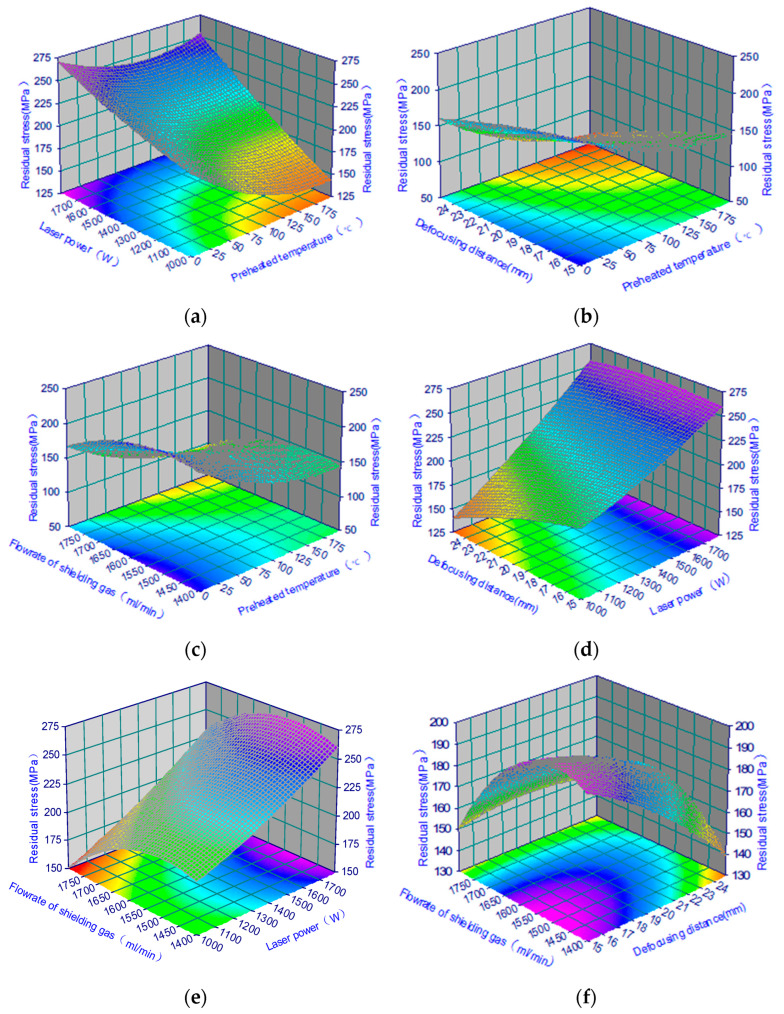
Contour plots of the effect on residual stress for the second order model. (**a**) The surface plot of preheated temperature and laser power on residual stress. (**b**) The graph of preheated temperature and defocusing distance on residual stress in the WC(Co,Ni) welds. (**c**) Surface plane plot of residual stress in the preheated temperature and flow rate of shielding gas. (**d**) Surface plot of residual stress in the defocusing distance and laser power. (**e**) The effect of the flow rate of shielding gas and laser power on residual stress. (**f**) Saddle contour plots in the flow rate of shielding gas and defocusing distance on residual stress.

### 3.5. Effect of Control Factors on Residual Stress Properties

The experimental results were analysed using analysis of variance (ANOVA), focusing on the sources of variation in residual stress in WC(Co,Ni) coatings. Based on ANOVA, it was determined which factors were the most important ones for residual stress. Therefore, these important factors can be monitored very carefully in the simulated results of residual stress in order to obtain robust results. As shown in Table 3, the important control factors include preheated temperature (D), laser power (E), defocusing depth (G), and flow rate of shielding gas (H). The secondary control factors are (A) substrate, (B) rate of Co%, (C) rate of Ni%, and (F) scanning speed, which exhibit much weaker effects on the variance. Apparently, laser power is the largest contributor to residual stress characteristics, accounting for 66.21% of the total. This is followed by preheating temperature (12.07%), defocusing depth (6.47%), and shielding gas flow rate (5.74%). Together, these factors account for 90.49% of the total. As a result, these four parameters are representative of all parameters that can be used for further in-depth analysis of the residual stress. The above-mentioned important parameters are further analysed by the response surface methodology.

### 3.6. Construction of Empirical Models

Table 1 shows the parametric design and observed responses based on Taguchi’s design, which shows the residual stress response of the laser-melted weld. Based on the ANOVA in Taguchi’s design, the important parameters of preheating temperature (D), laser power (E), defocusing depth (G), and shielding gas flow rate (H) were included in the regression analysis of residual stress by laser cladding. SPSS 22 was used to construct and analyse the experimental data for this design, which makes use of regression modelling. Table 1 presents the design variable representations in natural units. A model containing a quadratic term has been used in the response surface methodology.

Y = −563.727121 − 0.756609D − 0.175676E − 22.983093G + 1.372029H + 0.000320DE − 0.014414DG −  0.000043DH + 0.006460EG + 0.000047EH + 0.013667GH + 0.001872D^2^ + 0.000031E^2^ − 0.194505G^2^ −  0.000542H^2^    Adjust *R*^2^ = 0.841 (5)

Three important parameters, preheated temperature, laser power, defocusing depth, and flow rate of shielding gas, were selected based on Table 1 after ANOVA of Taguchi’s design. The experimental data were fitted using the above model. The fitting equation of the RSM model was calculated using Equation (4), while the residual stresses were determined using Equation (5). As shown in Table 4, the ANOVA results indicated that there was a significant difference between the linear, quadratic, and interaction models, with ‘Prob > F’ values of 0.0000508, 0.004006, and 0.00621, respectively. These values are all smaller than 0.05, indicating that the interaction regression model is significant. In addition, the adjusted R^2^ values of the regression coefficient of determination for the linear, interaction and quadratic models were 0.779, 0.822, and 0.841, respectively. However, the higher values of the adjusted coefficient of determination for the quadratic model suggest that there is higher significance for these models. The selected quadratic prediction model for residual stress (Y) is presented in Table 4, along with its fitting statistics. The model showed good agreement between the experimental and predicted values of residual stress yield, with only 15.8% of the total variance not explained by it. This was indicated by an analysis of variance through the ANOVA coefficient (R^2^ = 0.841). By performing multiple regression analysis on the experimental data, the relationship between the response variable and the test variable is shown in Equation (5). Its coefficient of regression is on the left side of Table 5. Based on the information obtained above, a secondary model was successfully constructed on the basis of the regression model. Furthermore, a three-contour model of the residual stress behaviour was further explored.

### 3.7. Effects of Variables on Modelling of Residual Stresses

To understand how the stresses in the WC/Co/Ni deposits behave, we need to show three-dimensional pictures of the second-order functions used in Equation (5). As shown in Figure 10, the second-order function is displayed. This figure shows a three-dimensional contour plot of the residual stress yield response surface under significant control factors. The four most important factors were selected through an analysis of variance (ANOVA) of the eight factors. The contour plot of the quadratic model is shown in Figure 10. Two variables were held at fixed low levels. The other two varied within the experimental range. The shape of the lines on the 3D pictures of the second-order functions is almost the same as the shape of the saddle-shaped patterns. Also, all of the plots are in the saddle-shaped pattern. This indicates that there is a significant interaction between the variables. Figure 10a shows a rough graph of the effect of preheating temperature and laser power on residual stress. The results demonstrate that an increase in laser power significantly increases residual stress. This occurs when the preheating temperature is within the range of 25–200 °C. The results also demonstrate that when the preheated temperature reaches 200 °C at a lower power of 1000 W, the WC results in less residual stress in the cladding zone. The saddle contour in Figure 10b indicates that the residual stress on the coating’s surface is smaller in the pink area when the defocusing distance is 25 mm, and the preheating temperature is 200 °C. It was mentioned above that laser power is an important factor affecting residual stress, and must be properly controlled. The pattern in Figure 10c is similar to that in Figure 10b, in which the residual stress fluctuates between 100 and 150 MPa. A pattern of saddles is evident in its contour plot. The residual stress area in the upper left corner of the two-dimensional plane in Figure 10c is noticeably lighter in colour. This indicates that Figure 10c exhibits higher stress behaviour. As can be seen from the 3D saddle contour plot in Figure 10d, the effect of laser power on residual stress increases remarkably from 130 MPa to 260 MPa when the defocusing distance remains within the 15–25 mm range. In short, the defocusing distance has much less influence on the residual stress than the laser power. It is clear that the pink area in the picture shows lower residual stress. Similarly, the residual stress is approximately 140 MPa when the defocusing distance is 25 mm, and the laser power is 1400 W. As shown in Figure 10e, there is a three-dimensional photograph, along with a two-dimensional contour plot. These illustrate the saddle pattern of residual stress. The stress is indicated by the function of laser power and shielding gas flow rate. The pattern in Figure 10e is similar to that in Figure 10d. When the shielding gas flow rate increases from 1400 mL/min to 1800 mL/min, the residual stress decreases from 275 MPa to 150 MPa. The effect of defocus distance and shielding gas flow rate on residual stress is illustrated in Figure 10f. This figure shows that, when the defocus distance varies from 15 mm to 25 mm, the flow rate of the shielding gas significantly affects residual stress. However, the residual stress fluctuates greatly. The graph shows that the flow rate of shielding gas is 1600 mL/min. The residual stress is higher at this flow rate. The figure above shows a saddle shape with high stress values in the middle and low stress values on both sides. Overall, the laser power and shielding gas flow rate have a greater effect on residual stress than the preheating temperature and defocusing distance. This is consistent with the results in Table 3. This study used contour maps to identify optimal areas within 3D images. These maps clearly demonstrate the relationship between residual stress and key factors. They also provide useful information about the cracks generated in the coating.

### 3.8. Analysis of Model Confirmation Experiments

**Figure 11:**
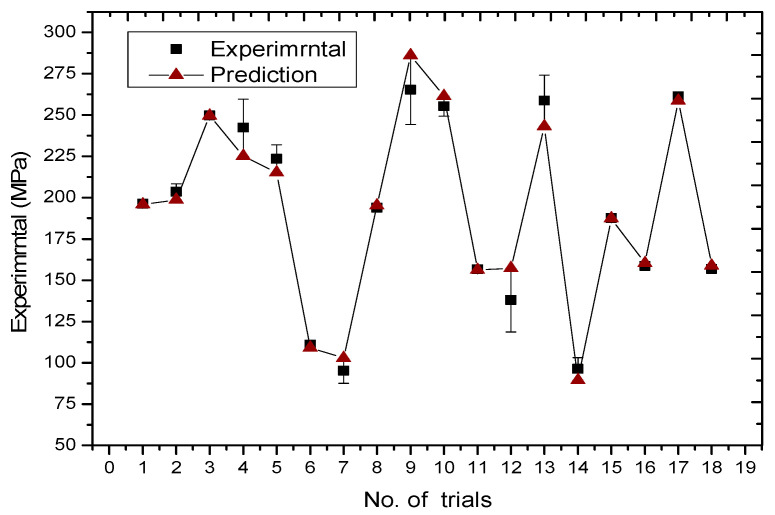
The distributions of the experimental and predicted patterns with the resulting standard errors.

The performance is predicted by the parameters provided by the model, which validates the model. This provides a better understanding of how the parameters affect residual stress behaviour. As shown in Table 3, the model was analysed using ANOVA. Four important factors were chosen because they explained 90.49% of the total variation. These were the preheating temperature, laser power, defocusing distance, and shielding gas flow rate. These factors were applied when building the model and making predictions. The experimental and quadratic model predictions were compared. This was performed in terms of validating the predictive ability of the model. The distributions of the experimental and predicted patterns are shown in Figure 11. The resulting errors in the predictions of the quadratic model are also shown. A comparison of all the experiments was made, and the results were then analysed. Most of the predictions were closer to the experimental values, except for trials 4, 9, 12, and 13, where the errors were greater than 10%. This indicates that the model is reliable. However, smooth fluctuations in the distribution of prediction errors for the quadratic model have been observed. The mistakes made by the model are no more than twice the standard deviation of the experimental values. This means that the predicted values are much closer to the experimental values. This suggests that the model is reliable. In addition, the above graph shows that the lower residual stress of about 108 MPa can be obtained by using a preheated temperature of 200 °C. It also shows that a laser power of 1000 W is needed. In addition, the defocusing distance should be 25 mm. The flow rate of the shielding gas should be 1800 mL/min. The experiment was conducted under the above-mentioned conditions. The results obtained from the experiment were then used to perform three confirmations. These produced respective values of 109 MPa, 97 MPa, and 112 MPa. The average value of these three values was 106 MPa. The predicted values are very close to the experimental values. This demonstrates that the model is reliable. Consequently, it can be concluded that the developed quadratic model can effectively forecast the residual stress behaviour of laser-coated WC(Co,Ni) welds.

## 4. Concluding Remarks

This paper addresses the optimisation of response surface methodology based on finite element analysis for laser melting highly hardened WC(Co,Ni) coatings, with the aim of enhancing the efficiency and precision of the process. The temperature and stress fields of the coating were investigated by simulations based on finite element analysis. A thermally coupled model was developed. This was performed to simulate the temperature field with a Gaussian distribution for the purpose of further weld stress calculations. Additionally, the residual stresses were modelled, and the predicted values were obtained using an RSM-based FEA model. The following conclusions are drawn from this study:

1. A numerical model of laser-melted WC(Co,Ni) welds under continuous loading of a moving laser spot has been established using the parametric design language of the ANSYS software. This model enables the patterns of distribution of the temperature and stress fields during coaxial laser welding to be derived.
2. The white areas within the melting zone of WC(Co,Ni) are dominated by carbides containing over 80% tungsten, while the carbon content is around 1.5–3.0%. In addition, a variety of carbide forms have been found in the large area of the melting zone, including dendritic, strip-like, leaf-like, net-like, and smaller clustered structures.
3. The results of the ANOVA analysis based on the experimental design showed that the effects of the four variables on residual stress were highly significant. Notably, the factors of preheating temperature, laser power, defocusing distance, and shielding gas flow rate accounted for 90.46% of the total variance.
4. The effects of key factors such as preheating temperature, laser power, defocus distance, and protective gas flow on residual stress are shown in the three-dimensional contours. Clearly, the factors shown in the figure can clearly explain the effect of residual stress.
5. The average error of the quadratic function was found to be 6.52% when a comparison of all the experiments was made. This suggests that the predicted values are very close to the experimental values. This indicates that the model is credible.

## Figures and Tables

**Table 1:** An experimental design by Taguchi for 18 runs of simulations of FSM for residual stress and S/N calculations for control factors and levels.

No. of Trials	Control Factors and Levels							
	Substrate	Ratio of Co (%)	Ratio of Ni (%)	Preheated Temperature (°C)	Laser Power (W)	Scanning Speed (mm/s)	Defocusing Distance (mm)	Flow Rate of Shielding Gas (mL/min)
1	#45	0	0	25	1000	2	15	1400
2	#45	0	10	100	1400	4	20	1600
3	#45	0	20	200	1800	6	25	1800
4	#45	10	0	25	1400	6	25	1600
5	#45	10	10	100	1800	2	15	1800
6	#45	10	20	200	1000	4	20	1400
7	#45	20	0	100	1000	4	25	1800
8	#45	20	10	200	1400	6	15	1400
9	#45	20	20	25	1800	2	20	1600
10	#40Cr	0	0	200	1800	4	15	1600
11	#40Cr	0	10	25	1000	6	20	1800
12	#40Cr	0	20	100	1400	2	25	1400
13	#40Cr	10	0	100	1800	6	20	1400
14	#40Cr	10	10	200	1000	2	25	1600
15	#40Cr	10	20	25	1400	4	15	1800
16	#40Cr	20	0	200	1400	2	20	1800
17	#40Cr	20	10	25	1800	4	25	1400
18	#40Cr	20	20	100	1000	6	15	1600

**Table 3:** An analysis of variance based on the experimental design for WC(Co,Ni) welds.

Control Factors	Sum of Squares	Degrees of Freedom	Mean Square	F-Value	Percent Contribution
A	1.323	1.0	1.323	0.542	0.89
B	3.222	2.0	1.611	0.660	2.17
C	1.419	2.0	0.710	0.291	0.96
D	17.917	2.0	8.958	3.671	12.07
E	98.303	2.0	49.152	20.144	66.21
F	3.262	2.0	1.631	0.669	2.20
G	9.610	2.0	4.805	1.969	6.47
H	8.529	2.0	4.264	1.748	5.74
Error	4.880	2.0	2.440	1.000	3.29
Total	148.466	17.0	8.733	3.579	100.00

**Table 4:** Analysis of variance of response of wear volume based on linear, interaction, and quadratic functions.

Source	Degree of Freedom	Sum of Squares	Mean Square	F-Test	Prob < F	Adjust-R^2^
Linear model	4	47,617.08	11,904.27	16.00383	6.08 × 10^−5^	0.779
Interaction model	10	53,161.47	5316.147	9.020209	0.004006	0.822
Quadratic model	14	55,681.99	3977.285	7.434195	0.00621	0.841

**Table 5:** The estimated coefficient of regression and *p*-value for the quadratic model.

Source	Quadratic Model			
	Coefficient Estimate	Standard Error	*t*-Statistical	Prob > F
Intercept	−563.727	936.196	−0.602	0.590
D	−0.757	1.283	−0.590	0.597
E	−0.176	0.361	−0.486	0.660
G	−22.983	30.477	−0.754	0.506
H	1.372	1.078	1.272	0.293
DE	0.000	0.000	0.977	0.401
DG	−0.014	0.026	−0.551	0.620
DH	0.000	0.001	−0.066	0.951
EG	0.006	0.006	1.121	0.344
EH	0.000	0.000	0.336	0.759
GH	0.014	0.011	1.222	0.309
D^2^	0.002	0.002	1.131	0.340
E^2^	0.000	0.000	0.404	0.714
G^2^	−0.195	0.499	−0.390	0.723
H^2^	−0.001	0.000	−1.759	0.177

## Data Availability

The raw data supporting the conclusions of this article will be made available by the authors on request.
